# Locally administered heparin-binding epidermal growth factor-like growth factor reduces radiation-induced oral mucositis in mice

**DOI:** 10.1038/s41598-020-73875-7

**Published:** 2020-10-15

**Authors:** Jing Chen, Laurent A. Bekale, Kelly M. Khomtchouk, Anping Xia, Zhixin Cao, Shoucheng Ning, Susan J. Knox, Peter L. Santa Maria

**Affiliations:** 1 Department of Otolaryngology, Head and Neck Surgery, Stanford University, 801 Welch Road, Stanford, CA 94305-5739 USA; 2 Department of Pathology, Shandong Provincial Hospital Affiliated to Shandong First Medical University, Jinan, 250021 Shandong China; 3 Department of Radiation Oncology, Stanford University, Stanford, CA 94305 USA

**Keywords:** Cancer, Drug discovery

## Abstract

Oral mucositis refers to lesions of the oral mucosa observed in patients with cancer being treated with radiation with or without chemotherapy, and can significantly affect quality of life. There is a large unmet medical need to prevent oral mucositis that can occur with radiation either alone or in combination with chemotherapy. We investigated the efficacy of locally administered heparin-binding epidermal growth factor-like growth factor (HB-EGF), a potent epithelial proliferation and migration stimulator of the oral mucosa as a potential therapy to prevent radiation induced oral mucositis. Using a single dose (20 Gy) of radiation to the oral cavity of female C57BL/6 J mice, we evaluated the efficacy of HB-EGF treatment (5 µl of 10 µg/ml) solution. The results show that HB-EGF delivered post radiation, significantly increased the area of epithelial thickness on the tongue (dorsal tongue (42,106 vs 53,493 µm^2^, p < 0.01), ventral tongue (30,793 vs 39,095 µm^2^, *p < 0.05)) compared to vehicle control, enhanced new epithelial cell division, and increased the quality and quantity of desmosomes in the oral mucosa measured in the tongue and buccal mucosa. This data provides the proof of concept that local administration of HB-EGF has the potential to be developed as a topical treatment to mitigate oral mucositis following radiation.

## Introduction

Oral mucositis is a debilitating complication of radiation and chemoradiation treatment for head and neck cancer, with an incidence of approximately 80%, largely due to radiation fields that include the salivary glands and/or the oral mucosa. Annually, there are approximately 400,000 cases of treatment-induced oral mucositis in the United States^1^. Severe mucositis, as defined by Radiation Therapy Oncology Group Criteria grade III or above, occurs with an incidence of at least 60% in head and neck radiation^2^. When it occurs, it may result in treatment interruption or even early termination of treatment, which can have an adverse effect on outcome^3^.

The pathogenesis of oral mucositis occurs in phases^4^. The initiation phase is collateral breaking and damage to DNA, a necessary effect required for cancer cell death. A message generation phase then occurs are a multitude of pro-inflammatory cytokines are released. The tissue then enters a signal amplification phase particularly in response to TNFα activation of ceramide and caspase pathways. An ulceration phase then occurs with mucosal breach and secondary infection. This can be extremely painful, both during and for weeks following therapy^3–5^. Finally a healing phase occurs with cell regeneration. The oral mucosa is particularly susceptible because of a rapidly replicating basal cell layer highly susceptible to radiation or chemotherapeutic injury, leading to decreased cell regeneration, epithelial thinning, and subsequent denudation^3^.

There are limited treatment options for this indication^6^, which include moisturizers, oral care, analgesia, anti-inflammatories and antibiotics and antifungal medications^2^. Palifermin or Recombinant Keratinocyte Growth Factor (KGF) (Kepivance, Amgen, Thousand Oaks, USA) was a treatment option until recently when it was withdrawn from the market in 2017. Palifermin was delivered systemically, and despite good efficacy and demonstrated costs savings of $3595 per patient, it was poorly adopted for the treatment of head and neck cancer as the cost and patient inconvenience were thought to outweigh the benefits achieved^7^. The development and initial success of Palifermin showed that epithelial stimulation was an excellent approach to preventing oral mucositis. However, its failure showed that a more convenient method of administration was needed. KGF is known to have stability problems, particularly in the harsh oral environment, where peptides are exposed to salivary enzymes, and therefore is not an ideal candidate for local administration^8^.

Given results to date with a variety of approaches to prevent and treat radiation-induced mucositis, the optimal strategy appears to be stimulation of mucosal basal cell regeneration using a local delivery method. We therefore studied the effect of a potent stimulator of keratinocyte proliferation and migration, Heparin Binding Epidermal Growth Factor Like Growth Factor (HB-EGF), since we had previously shown that HB-EGF had efficacy in oral wound healing, with demonstrated stability in saliva, and expected negligible systemic exposure when applied locally^9–11^. Our primary objective was to test the hypothesis that HB-EGF would have a significant beneficial effect for the prevention of radiation-induced oral mucositis in a clinically relevant animal model.

## Results

### Local administration of HB-EGF reduces the effects of radiation and enhances epithelial proliferation on tongue mucosa following radiation injury

**Figure 1:**
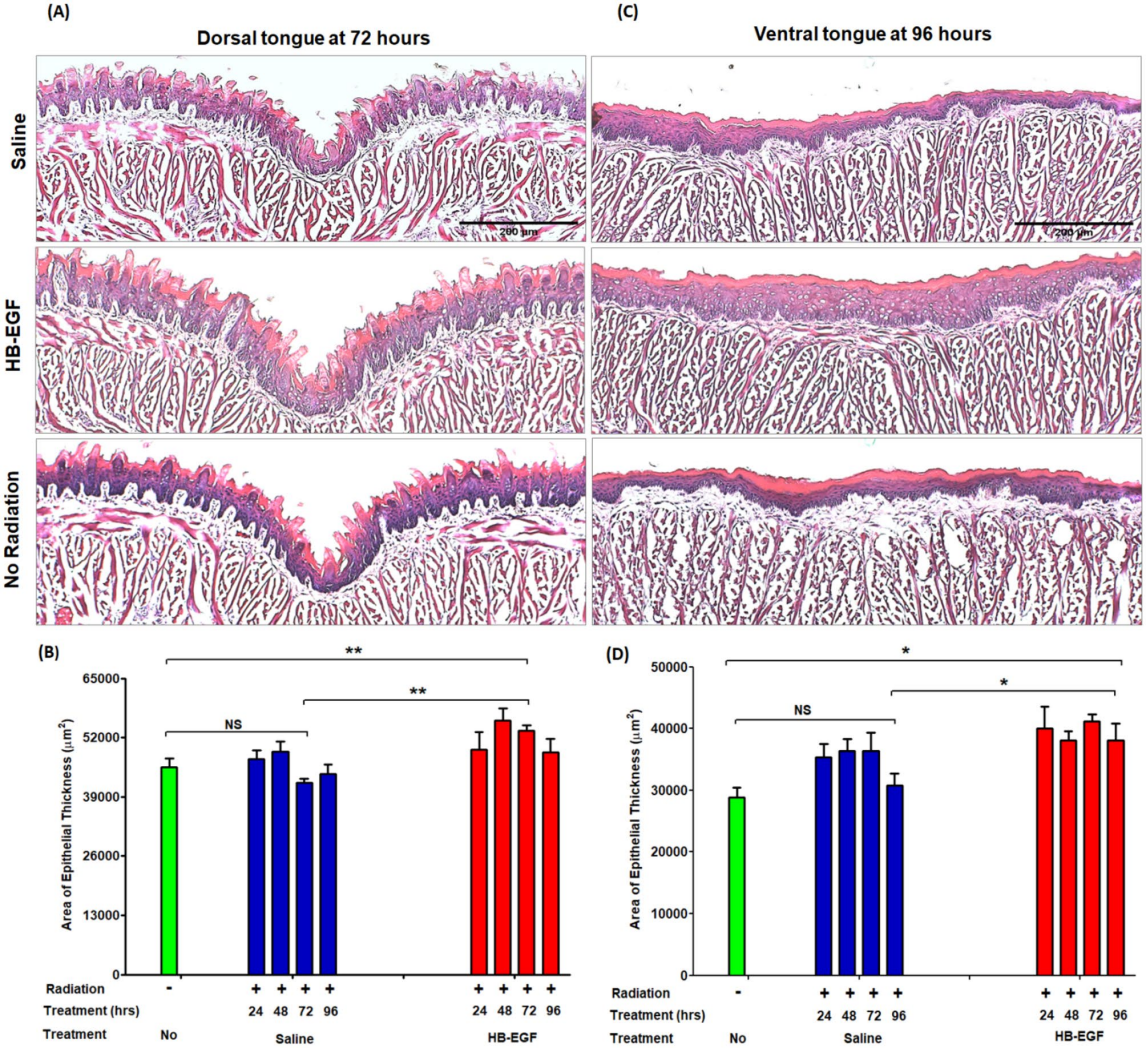
Beneficial effect of HB-EGF treatment on epithelial morphology in tongue. Area of epithelial thickness were assessed over a time course of 96 h of single-dose radiation of 20 Gy with daily doses HB-EGF of 0.05 µg/5 µl/injection locally into tongue. (**A**) and (**C**) Hematoxylin and eosin stained Histologic photographs (× 100) of dorsal (72 h) and ventral sides (96 h) of tongue specimens after radiation and saline or HB-EGF treatment. (**B**) and (**D**) Statistics graphs of area of epithelial thickness in tongue treated with saline (blue) and HB-EGF (red) after radiation. *P < 0.05, **P < 0.01, n = 4–7, scale bar 200 µm.

There was no evidence of tissue injury from the local delivery of the HB-EGF, with no difference in histological findings between animals treated with the non-radiated control group (Fig. 1A,C). Local administration of saline (vehicle control) in irradiated animals had no significant effect on epithelial thickness on the dorsal and ventral surface of tongue compared to radiation (Fig. 1B,D). In contrast, following HB-EGF treatment, the area of epithelial thickness increased significantly after irradiation compared to no radiation (on the dorsal tongue at 72 h: 45,571 vs 53,493 µm^2^, **p < 0.01, and ventral tongue at 96 h: 28,841 vs 39,095 µm^2^, *p < 0.05) (Fig. 1B,D). Furthermore, HB-EGF treatment significantly increased the area of epithelial thickness on the dorsal (42,106 vs 53,493 µm^2^, **p < 0.01) and ventral surfaces of the tongue (30,793 vs 39,095 µm^2^, *p < 0.05) compared to vehicle control in irradiated animals (Fig. 1B,D). Altogether, this data demonstrates the ability of post-irradiation HB-EGF to mitigate radiation-induced damage and facilitate the healing process on the tongue.

We next asked whether the combination of pre and post-treatment could further increase the therapeutic efficacy of HB-EGF of oral cavity irradiation using the dorsal region of the tongue as a representative specimen. Comparison of the increases in epithelial thickness in the ventral region (96 h) revealed that pre-treatment with HB-EGF provided no additional benefit (supplementary information Fig. S1). Thus, post-treatment with HB-EGF was chosen as the best mode of treatment to reduce radiation-induced oral mucositis in subsequent experiments.

**Figure 2:**
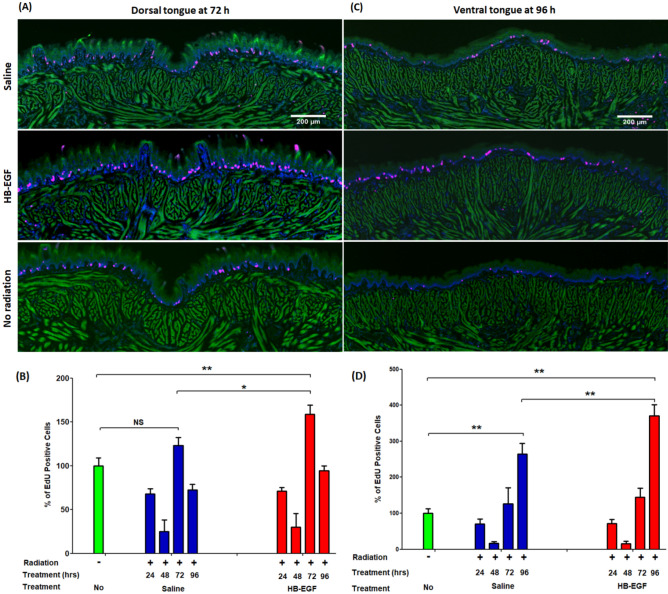
Effect of HB-EGF treatment on epithelial cell proliferation (EdU positive cells-red color) and expression of EGFR (Green color) by immunofluorescence staining (× 50) in tongue. EdU positive cells (red) were counted through whole epithelia layer in the micrographs and calculated over a time course of 96 h of single-dose radiation of 20 Gy with daily doses HB-EGF of 0.05 µg/5 µl/injection locally into tongue. (**A**) and (**C**) Immunofluorescence stained EdU positive cells' histologic photographs of dorsal side (72 h) and ventral side (96 h) of tongue specimens after radiation and saline or HB-EGF treatment. (**B**) and (**D**) Statistics graphs of the percentage of EdU positive cells to no radiation group in tongue treated with saline (blue) and HB-EGF (red) after radiation. *P < 0.05, **P < 0.01, n = 4–7, scale bar 200 µm.

To further demonstrate the beneficial role of HB-EGF treatment on the healing process of the tongue, we measured epithelial cell proliferation using incorporation of either 5-ethynyl-20-deoxyuridine (EdU). This technique precludes measurement of false positive responses, since inflammation itself may cause an increase in epithelial thickness^12,13^. Histological immunofluorescence staining of the dorsal and ventral tongue shows the percentage of EdU positive cells in the Fig. 2. Following radiation-induced epithelial damage, the level of EdU positive cells generally decreases during the first 24 h and begins to increase after 48 hours^14,15^. With saline treatment, there is significant change in EdU staining only on the ventral surface of tongue (96 h) compared to the negative control (100 vs 265%, **p < 0.01) (Fig. 2C,D). HB-EGF local injection dramatically increased the EdU positive cells in the ventral surface of the tongue (96 h) compared to vehicle control (265 vs 371, **p < 0.01) (Fig. 2D). EdU staining was also increased in the dorsal tongue epithelium of HB-EGF treated mice (72 h) after radiation compared to the vehicle control treated animals (123 vs 159%, *p < 0.05) (Fig. 2B). Furthermore, epithelial cell proliferation occurs more rapidly in the dorsal (maximum of the total number of positive cells at 72 h) than in the ventral side of the tongue (maximum of the total number of positive cells at 96 h), as demonstrated by the EdU staining in both the saline and HB-EGF treated groups (Fig. 2B,D). Overall, this experiment confirms that the increase of area of epithelial thickness shown in Fig. 1 is due to epithelial cell proliferation induced by HB-EGF.

### Local administration of HB-EGF reduces the effects of radiation and enhances epithelial proliferation on the buccal mucosa following radiation injury

**Figure 3:**
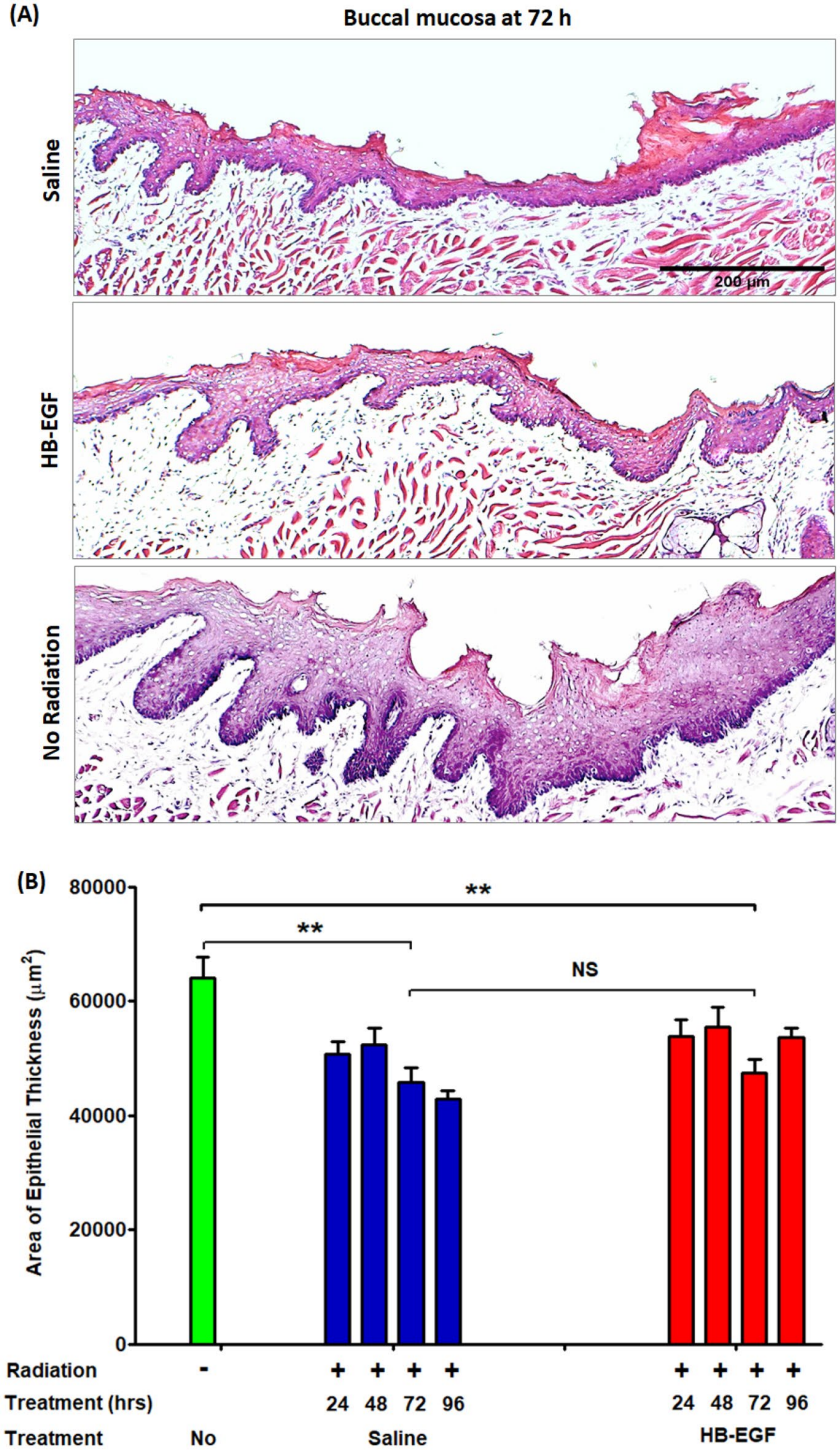
Beneficial effect of HB-EGF treatment on epithelial morphology in oral buccal. Area of epithelial thickness were assessed over a time course of 96 h after single-dose radiation of 20 Gy with daily doses HB-EGF of 0.05 µg/5 µl/injection locally to right side of buccal. (**A**) Hematoxylin and eosin stained Histologic photographs (× 100) of buccal specimens at 72 h after radiation and saline or HB-EGF treatment. (**B**) Statistics graphs of area of epithelial thickness in buccal mucosa. **P < 0.01, n = 4–7, scale bar 200 µm.

**Figure 4:**
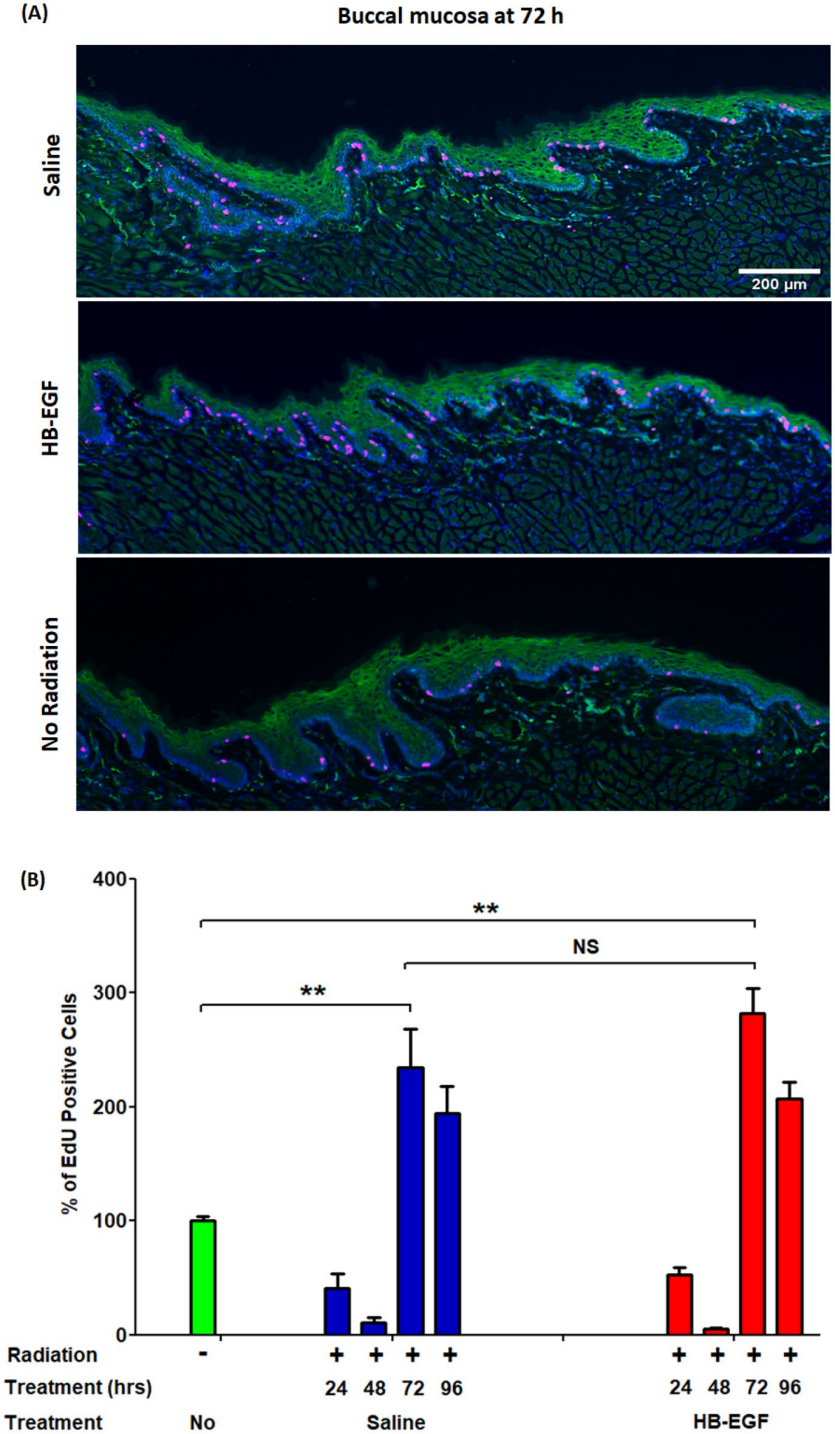
Effect of HB-EGF treatment on epithelial cell proliferation (EdU positive cells—red color) and expression of EGFR (green color) by immunofluorescence staining (× 50) in buccal mucosa. EdU positive cells (red) were counted through whole epithelial layer in micrographs and calculated over a time course of 96 h after single-dose radiation of 20 Gy with daily doses HB-EGF of 0.05 µg/5 µl/injection locally to right side of buccal mucosa. (**A**) Immunofluorescence stained EdU positive cells' Histologic photographs of buccal mucosa specimens at 72 h after radiation and saline or HB-EGF treatment. (**B**) Statistics graphs of the percentage of EdU positive cells to no radiation group (100%) in buccal mucosa treated with saline (blue) and HB-EGF (red) after radiation. **P < 0.01, n = 4–7, scale bar 200 µm.

Similar to the tongue specimens, there was no evidence of tissue injury due to the injections themselves (Fig. 3A,B). After radiation in the saline treatment group, there was a significant decrease in the area of epithelial thickness, which reached a minimum at 72 h compared to the negative control (45,837 vs 64,023 µm^2^, **p < 0.01) (Fig. 3B). HB-EGF solution injected intralesionally in each buccal mucosa increased the area of epithelial thickness, but was not significantly different compared to the saline group (Fig. 3B). Histological immunofluorescence staining of the buccal mucosa is shown as the percentage of EdU positive cells in the Fig. 4. 48 h after irradiation, there was a progressive decrease in EdU positive cells in both saline (100 vs 11%, **p < 0.01) and HB-EGF treated groups (100 vs 5.4%, **p < 0.01). The EdU staining in the buccal mucosa is similar to that of the dorsal surface of the tongue. HB-EGF local injection slightly increased the EdU positive cells compared to saline (Fig. 4). Again, this experiment demonstrated that HB-EGF-induced epithelial cell proliferation is responsible for the observed increase in the area of epithelial thickness in treated buccal mucosa (Fig. 4).

### Local administration of HB-EGF did not change EGFR expression in epithelium of tongue and buccal mucosa

In order to characterize the pattern of expression of EGFR after HB-EGF treatment with or without radiation, we performed immunofluorescence staining of EGFR (green color) in tongue tissue (Fig. 2A,C) and buccal mucosa (Fig. 4A). EGFR was evenly expressed in epithelial and muscle of tongue and epithelium of buccal mucosa. There was no differences noted by blinded observers between radiation and non-radiation treatment or between saline and HB-EGF treated samples. Of note is that the EGFR immunostain broadly stains all subtypes of EGFR (HER1,2,3,4) and there currently is not available method to perform immunohistochemistry on EGFR subtypes.

### Local administration of HB-EGF improves oral desmosome quality and quantity following radiation injury

**Figure 5:**
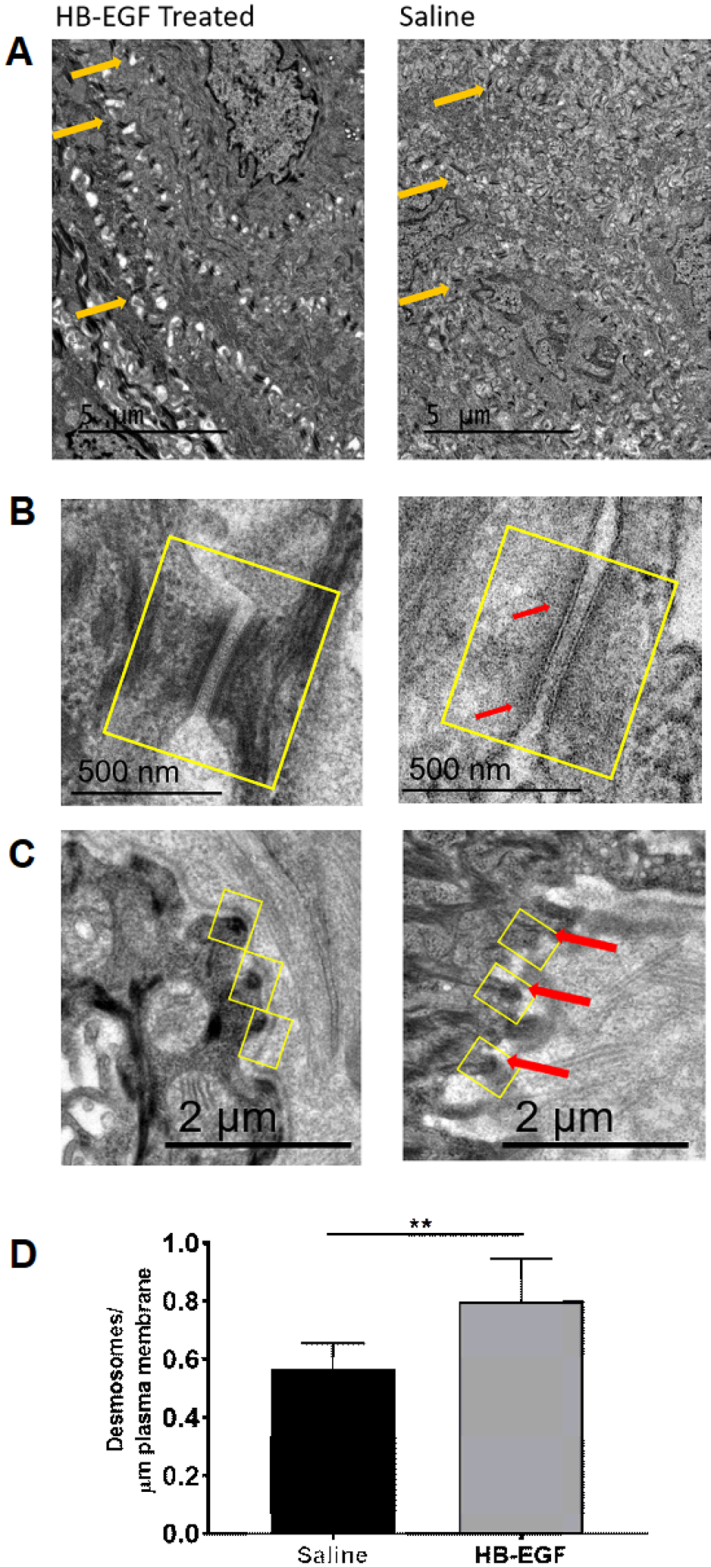
HB-EGF increases desmosome frequency and maintains desmosome structure after radiation. (**A**) Representative images of transmission electron microscopy of HB-EGF and saline treated tongue showing cross sections of epithelial junctions. Orange arrows point to desmosomes on the plasma membrane. (**B**) The yellow box indicates the desmosome junction. Red arrows illustrate disruption of the proteins in the intermediate filament network in vehicle-treated animals. (**C**) Cross sections of hemi-desmosomes. The yellow box indicates the hemi-desmosome junction. Red arrows illustrate disruption of the proteins in the intermediate filament network in vehicle-treated animals. After HB-EGF treatment there was a visible increase in the density of cell to extracellular matrix associations. (**D**) HB-EGF treatment of irradiated mice resulted in an increase in the number of desmosomes per micrometer length of plasma membrane of epithelial cells in the ventral tongue. Desmosomal quantification was performed on electron micrographs by three blinded reviewers. One to four micrographs of three animals per treatment group were assessed. **P < 0.01 compared to control.

Tongue from Irradiated vehicle and HB-EGF treated mice were compared post-irradiation using transmission electron microscopy. Cross sections of epithelial junctions from these mice are shown in Fig. 5. Desmosomes, proteins located in the intermediate filaments and hemidesmosomes, per micrometer length of plasma membrane, were increased in tongue epithelium of HB-EGF treated mice after radiation compared to the vehicle control (0.568 vs 0.799, **p < 0.01). Interobserver reliability was found to have an average coefficient of variation under 30%.

## Discussion

### Oral mucositis model in mice

The radiation model that we choose is one that has been previously validated as representative of the human histological situation^16^. It is also the same model chosen in the pre-clinical validation of efficacy for recombinant KGF prior to its approval for clinical trials. Interestingly, rodents do not develop oral mucosits in the same way as humans, without frank open lesions. When the oral changes are severe enough they quickly progress towards weight loss and death. If this model is left untreated, the mice progress towards death within 8 days^17^. It has been previously validated that decreasing epithelial thickness early following radiation, increasing epithelial division early and desmosome quality and quantity can act as oral mucositis biomarkers for the development of oral mucositis^16^. The clinical efficacy gives us confidence that these biomarkers are relevant to the human situation.

### Locally administered HB-EGF is a promising candidate to decrease radiation-induced oral mucositis

In the present study we show that locally administered HB-EGF significantly decreased radiation-induced oral mucositis in an animal model previously shown to be predictive for the translation of keratinocyte growth factor. We demonstrate this in a previously validated animal model of mucositis focusing on the buccal (non-keratinized) and the tongue (keratinized) areas^18–20^. We observed that HB-EGF delivered post radiation, significantly increased area of epithelial thickness, new epithelial cell division and increased quality and quantity of oral mucosal desmosomes. These histological biomarkers have been shown to be predictive in the development of oral mucositis^16^**.** Indeed, 24 h after radiation, inhibition of cell proliferation in the dorsal and ventral surfaces of the tongue are approximately 60%, 30% and 20%, respectively. We found that radiation causes greater injury in the buccal areas compared to the tongue. This is because non-keratinized oral tissues (buccal mucosa, lateral tongue, soft palate, floor of mouth) are more susceptible to oral mucositis than keratinized oral tissues^21^.

HB-EGF is from the EGF growth factor family but is structurally very different^22^. Unlike EGF which binds to all four receptor subtypes, HB-EGF has specific binding to the HER1 and HER4 receptors. HB-EGF additionally acts by EGFR-independent mechanisms ^23–25^. HB-EGF was chosen as a potential therapy for this indication given its ability to promote the wound healing process in multiple stages of wound healing, including granulation, re-epithelialization, tissue deposition, wound contracture, neovascularization, and remodeling^26–29^. We were also encouraged by the ability of HB-EGF to act on the oral mucosa and the demonstrated potential for translation^11,30^. We have previously shown that HB-EGF, not EGF has significantly greater ability to act on keratinocytes in vivo^9^. The newly formed epithelium treated with HB-EGF is also less likely to show delayed epithelial separation and sloughing. HB-EGF given locally following radiation improves epithelial coverage, and the keratinocyte connections are of greater quantity and quality to prevent future separation and breakdown^11^. In our study we did not detect a difference in EGFR expression pattern between HB-EGF treated and non-treated tissue. This could be because the method is unable to separate between EGFR receptor subtypes or that the EGFR independent mechanisms of HB-EGF could be important in this disease. Both KGF and HB-EGF look to act to reduce the impact of the ulceration phase by thickening the epithelium, improving the adhesion within the tight junctions and therefore prevent epithelial separation and breakdown. If breakdown does occur, they also have the effect of enhancing epithelial proliferation and migration to improve the healing phase.

### HB-EGF has advantages over KGF as a local therapy

Previous studies have confirmed the ability of HB-EGF to protect the intestines^31^, heart^32^, brain^33^ and kidneys^34^ from various forms of injury. Our recent work confirms the ability of HB-EGF to enhance epithelial repair in chronic tympanic membrane perforations^27^ and tonsillectomy wounds^11^.HB-EGF is more stable with favorable permeability compared to other proposed growth factor treatments^31^. As above, KGF has shown efficacy as a systemic therapy for oral mucositis, but due to poor stability, a short half-life, and low permeability it is not an ideal candidate for local therapy in the oral cavity^8^.

### Future directions

Although we have demonstrated that locally applied HB-EGF has potential utility for the prevention of radiation-induced oral mucositis, translation of this finding into clinical trials will require the development of a topical mucoadhesive drug delivery system. This system should be designed to enable prolonged retention at the site of application allowing for a therapeutic dose of HB-EGF to transfer into the mucosa. Evaluation and comparison of the efficacy of HB-EGF via topical solution is the subject of future studies. Before this treatment can be considered as a true solution for oral mucositis, it needs to demonstrate that it will not promote tumorigenicity in existing epithelial cells, further promote the growth of existing squamous cell carcinoma in the oral cavity or reduce the tumor sensitivity to adjuvant therapies. These risks are the focus of future investigations.

## Conclusion

This study demonstrates that locally administered HB-EGF post-irradiation significantly decreased histological findings associated with oral mucositis. HB-EGF could stimulate the proliferative response required for the regeneration of mucosal barrier function. These findings are clinically relevant, and suggest that HB-EGF is a promising candidate for local delivery in the oral cavity with near term translational potential.

## Materials and methods

### Animal cohorts

All animal work was approved by Stanford University’s Administrative Panel on Laboratory Animal Care. The 10 week-old female C57BL/6 J mice were purchased from Jackson Laboratories (Sacramento, CA) and housed in Stanford University’s animal resources facility according to standard guidelines in which food and water were provided ad libitum with the room maintained in 12-h dark/light cycles.

**Scheme 1:**
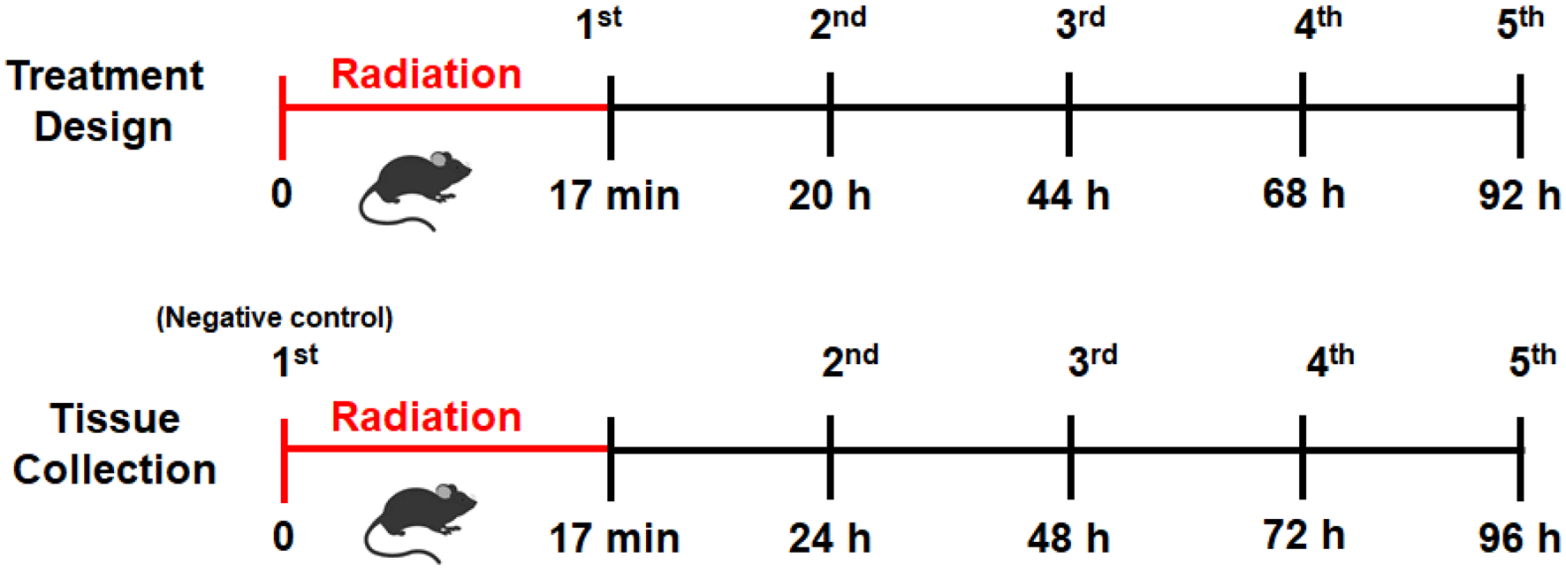
Cartoon showing the experimental design with topical HB-EGF treatment and tissue collection.

Sixty mice were randomized into 9 groups. Among them, 4 mice were allocated to the no radiation group and 7 mice per group for irradiation treatment groups. The study protocol including radiation, and daily treatment with vehicle (5 µl of saline) or HB-EGF (5 µl of 10 µg/ml) solution (Scheme 1).

### Radiation technique

Our oral mucositis radiation protocol is adapted from a validated model in mice, the same model that was used in the pre-clinical development of Keratinocyte Growth Factor (Palifermin, Kepivance, Amgen, Thousand Oaks, CA)^16,35^. This model has been validated as showing histological features similar to the human disease. Generation of single-dose radiation was done using a 0.5 mm Copper source (KIMTRON Inc, Model IC-225, Woodbury, CT, USA). Mice were anesthetized by intraperitoneal injection of Ketamine/Xylazine anesthesia cocktail (ketamine of 100 mg/kg plus xylazine of 10 mg/kg BW) and then placed headed to center into custom lead jigs on a hexagonal wood plate allowing protection of the whole body from the neck down with lead shielding, resulting in the exposure of the head only to radiation to a dose of 20 Gy at the condition of 225 kV, 13 mA and 50 cm of focus to skin distance (FSD), 6 mice each time.

### HB-EGF post treatment

Recombinant mouse HB-EGF was purchased from Prospec Bio (New Jersey, USA, Cat # CYT-068). It was prepared in saline to 1 mg/ml of stock solution, aliquoted to 100 μl/vial and stored at – 20 °C. The stock solution was freshly diluted to 10 µg/ml before use. Saline was chosen as the conferred vehicle.

After irradiation (day 0), mice were immediately treated HB-EGF solution with 0.05 µg/5 µl/injection or the same volume of saline at the right side of the buccal mucosa and the left side of tongue, respectively. From day 1 (first day after radiation) to 4, mice were anesthetized with isoflurane (5% induction and 2–2.5% maintaining concentration) under 1L/min oxygen flow and then injected with HB-EGF solution or the same volume of saline as above. Animals were injected intraperitoneally with 5-ethynyl-2′-deoxyuridine (EdU, ThermoFisher Scientific, Cat # A10044) by 25 mg/kg body weight 24 h before sacrifice. In all experiments, the mice were weighed daily and monitored closely. There were no unexpected animal deaths. Animal were euthanized with CO_2_ gas after 4 h of the last treatment. Immediately following sacrifice, the tongue and right buccal tissue were excised and fixed in 4% paraformaldehyde (PFA) for histological analysis followed by Optimal Cutting Temperature (OCT) embedding. Cross sections were then cut at 7 μm thickness from the anterior end of the tongue tip or buccal sulcus to the posterior end.

### HB-EGF combination treatment

For combination, post HB-EGF treatment was similar as above but pretreatment was performed daily for 3 days before radiation using the same HB-EGF concentration as post treatment.

### Histology, EdU detection, and immunofluorescence

For histological evaluation of tongue and buccal mucosa structure, hematoxylin and eosin-staining was performed on OCT-embedded tissues. Morphometry of epithelium was measured as area of epithelial thickness on the sections manually contoured using the Image J (NIH) analyzer^34^. The area of epithelia thickness in light micrograph images was measured from the basement membrane of the germinal layer to the interface with keratinized nucleated layer of the dorsal, ventral surface of the tongue and buccal mucosa around a whole image (× 100).

5-Ethynyl-2′-deoxyuridine (EdU) is a thymidine analogue which is incorporated into the DNA of dividing cells. For epithelial cell proliferation, EdU was detected using the Click-It EdU AlexaFluor 647 Imaging kit (ThermoFisher Scientific, Cat# C10340) according to the manufacturer's protocol. For epidermal growth factor receptor (EGFR) expression, EGFR immunofluorescence was performed. Using dual EdU and EGFR staining, EdU staining was performed first, and then EGFR immunostaining was done with formalin-fixed and OCT-embedded sections of mouse tongue or buccal mucosa. Followed by EdU detection, the slides were incubated in blocking solution (1% bovine serum albumin, 5% normal donkey serum, and 0.1% Triton X-100 in PBS) for 1 h at room temperature. After sucking the blocking solution, slides were incubated with primary antibodies (Abcam: rabbit monoclonal anti-EGFR antibi, catalog # ab40815, 1:300 in blocking solution) at 4 °C for overnight. After washing with 1X PBS for 3 times by 5 min, slides were incubated with secondary antibodies of Alexa Fluor 488 donkey anti-rabbit IgG (Life technologies, dilution of 1:500) for 1 h at room temperature, and then washed with 1× PBS for 3 times by 5 min each. Coverslips were then placed with Prolong Gold antifade reagent with DAPI (Life Sciences) to stain the nucleus as blue color. Only EdU positive staining cells were counted in the whole epithalial layer part of dorsal, ventral tongue and buccal mucosa in fluorescence micrograph images, and then calculated the percentage to no radiation group (100%). EGFR was presented as green color. Only EdU positive staining cells were performed quantitative analysis. EGFR was expressed evenly in epithelium and muscles part in tongue and epithelium in buccal mucosa, no quantitative results were reported. Area of epithelial thickness measurement and EdU positive cell counting were performed blindly by researchers.

### Desmosome assessment

Tongues were sectioned into 1 mm cubes at time of necropsy. Samples were fixed in 2% glutaraldehyde (EMS Cat# 16000)/4% paraformaldehyde (EMS Cat# 15700) in sodium cacodylate buffer (pH 7.3) (EMS Cat# 12300) for 24 h at room temperature and then stored at 4 °C. 1% Osmium tetroxide(EMS Cat# 19100) was added and samples were then allowed to warm to Room Temperature (RT) for 2 h rotating in a hood, washed 3X with ultra-filtered water, then en bloc stained in 1% Uranyl Acetate at RT 2hrs while rotating. Samples were then dehydrated in a series of ethanol washes for 30 min each at RT beginning at 50%, 70% EtOH then moved to 4 °C overnight. They were place in cold 95% EtOH and allowed to warm to RT, changed to 100% 2X, then Propylene Oxide (PO) for 15 min. Samples are infiltrated with EMbed-812 resin (EMS Cat# 14120) mixed 1:2, 1:1, and 2:1 with PO for 2 h each, leaving samples in 2:1 resin to PO overnight rotating at RT in the hood. The samples were then placed into EMbed-812 for 2–4 h, and then placed into molds w/labels and fresh resin, orientated and placed into 65 °C oven overnight.

Tongue was also cryosectioned to 1 micron thickness and stained with 0.1% toluidine blue. Selected areas were ultra-thin sectioned between 75 and 90 nm, picked up on formvar/Carbon coated slot Cu grids, and contrast enhanced with stained for 40seconds in 3.5% uranyl acetate in 50% Acetone and lead citrate staining (0.2%) for 6 min.

Using a JEOL JEM-1400 120 kV Transmission Electron Microscope, desmosome images were taken using a GatanOrius 832 4 k × 2.6 k digital camera with 9um pixel size. The representative TEM images of tongue surface show desmosome macrostructure. The number of desmosomes was counted per image by three blinded reviewers and the total perimeter of the plasma membranes within the image was measured to generate the perimeter length. Data were analyzed using a ratio paired t test.

### Statistical analysis

GraphPad Prism software (version 5.01) was applied for statistical analysis. Data are presented as mean ± standard deviation (SD) values (n = 4–7 per group). Comparisons were performed by ANOVA and two-tailed Student's t-tests. P value < 0.05 was considered a significant difference.

## Supplementary information

Supplementary file 1
