# Supplementary material for: Locally administered heparin-binding epidermal growth factor-like growth factor reduces radiation-induced oral mucositis in mice
Source: Sci Rep. 2020 Oct 15;10:17327. doi: 10.1038/s41598-020-73875-7 (PMC7567084; doi:10.1038/s41598-020-73875-7)
Supplement: Supplementary file 1 [file 41598_2020_73875_MOESM1_ESM.docx]

**Locally Administered Heparin-Binding Epidermal Growth Factor-Like Growth Factor Reduces Radiation-Induced Oral Mucositis in Mice**

Jing Chen^1^, Laurent A. Bekale^1٭^, Kelly M. Khomtchouk^1^, Anping Xia^1^, Zhixin Cao^1,2^, Shoucheng Ning^3^, Susan J. Knox^3^, and Peter L. Santa Maria^1٭^

^1^Department of Otolaryngology, Head and Neck Surgery, Stanford University, 801 Welch Road Stanford, CA 94305-5739, USA

^2^Department of Pathology, Shandong Provincial Hospital Affiliated to Shandong First Medical University, Jinan, Shandong 250021, China

^3^Department of Radiation Oncology, Stanford University, Stanford, CA 94305, USA

*email:[bekale20@stanford.edu](about:blank) and [petersantamaria@stanford.edu](about:blank)

Combination of pre and post-treatment do not increase the therapeutic efficacy of HB-EGF

**
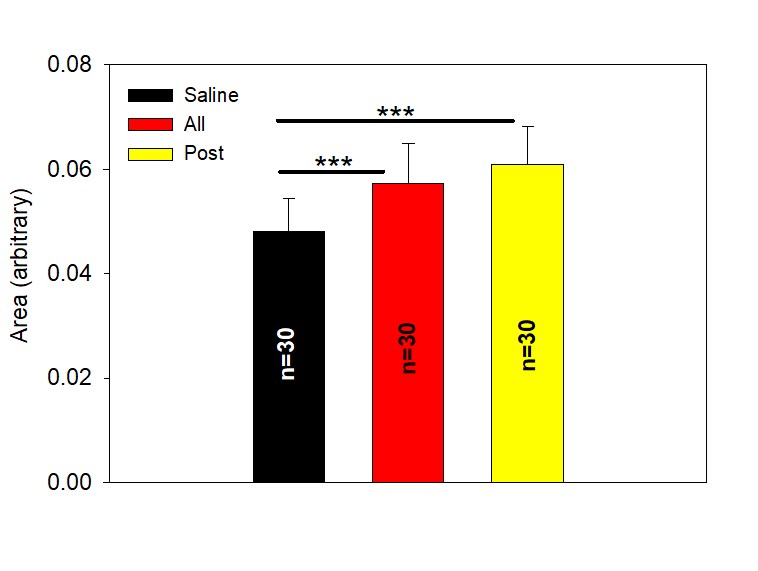
Supplementary information Fig. S1** Comparison of efficacy between post and combination (pre and post) HB-EGF treatment on epithelial morphology in tongue. Area of epithelial thickness were assessed at 96 hours of single-dose radiation of 20 Gy with daily doses HB-EGF of 0.05µg/5µl/injection locally into tongue.
